# Recent Advances in the Enantiomeric Separation of Tropane Alkaloids by Liquid Chromatography: Analytical Strategies and Applications in Pharmaceutical, Biological, and Food Samples

**DOI:** 10.3390/toxins18100455

**Published:** 2026-10-19

**Authors:** Fernando L. Vera-Baquero, Lorena González-Gómez, Isabel Sierra, Sonia Morante-Zarcero

**Affiliations:** 1 Departamento de Tecnología Química y Ambiental, E.S.C.E.T, Universidad Rey Juan Carlos, C/Tulipán s/n, 28933 Móstoles, Spain; fernando.vera@urjc.es (F.L.V.-B.); lorena.gonzalez@urjc.es (L.G.-G.); 2 Instituto de Investigación de Tecnologías para la Sostenibilidad, Universidad Rey Juan Carlos, 28933 Móstoles, Spain

**Keywords:** chiral analysis, hyoscyamine, atropine, chiral liquid chromatography, chiral stationary phases, sample preparation, racemisation, food contaminants

## Abstract

This review examines recent advances in the enantiomeric separation of tropane alkaloids (TAs), focusing on atropine, a racemic mixture of (+)- and (−)-hyoscyamine whose enantiomers differ significantly in pharmacological activity, with (−)-hyoscyamine being the more potent antimuscarinic agent. Enantiomeric purity and racemisation control are therefore essential in pharmaceutical formulations, biological samples, and food, where TAs are regulated contaminants due to their toxicological effects. The review covers analytical strategies reported from 2010 to 2026, with emphasis on high-performance liquid chromatography (HPLC), often coupled with tandem mass spectrometry (MS/MS) to improve sensitivity and selectivity. Polysaccharide-based chiral stationary phases operated in normal-phase or polar organic modes, usually with amine additives, represent the most widely employed approach. Protein-, cyclodextrin-, and antibiotic-based chiral selectors have also demonstrated successful enantiomeric resolution. Sample preparation is particularly challenging for food matrices, requiring efficient extraction and clean-up while preventing enantiomeric racemisation. Published strategies include solid-phase extraction (SPE), matrix solid-phase dispersion (MSPD), and QuEChERS protocols. Despite significant analytical advances, chiral analysis of TAs in foods remains underexplored. Recent studies have reported (−)-hyoscyamine concentrations exceeding regulatory limits in herbal infusions, underscoring the need for further research to support European Food Safety Authority (EFSA) recommendations and improve consumer risk assessment.

## 1. Introduction

Chirality is a fundamental stereochemical property arising from the three-dimensional arrangement of atoms in a molecule, resulting in an object that is not superimposable on its mirror image. Chiral molecules exist as two non-superimposable mirror-image forms known as enantiomers. Enantiomers possess identical physicochemical properties in achiral environments but differ in their interactions with chiral entities, including biological receptors, enzymes, and transport proteins. They also exhibit optical activity, rotating plane-polarised light by equal amounts in opposite directions. Accordingly, enantiomers are designated as dextrorotatory (+) or levorotatory (−), depending on whether the rotation is clockwise or counterclockwise, respectively. When both enantiomers are present in equal proportions, a racemic mixture, denoted as (±), is formed. Racemates are optically inactive because the opposite optical rotations of the two enantiomers cancel each other out [1].

The importance of chirality is particularly relevant in pharmaceutical, biological, and food-related compounds. Differences between enantiomers can have important implications for efficacy, safety, and risk assessment. In pharmaceutical applications, stereochemistry may significantly influence both pharmacodynamic properties and pharmacokinetic behaviour, including absorption, distribution, metabolism, and excretion. Consequently, one enantiomer may account for the desired therapeutic activity, whereas its counterpart may exhibit lower potency or different biological effects or contribute to adverse reactions [2,3].

In chiral drugs, the eutomer is responsible for the desired pharmacological effect, whereas the distomer may exhibit lower activity or contribute to adverse effects. As a result, chirality is a key consideration in drug development, since enantiomers can differ markedly in efficacy, metabolism, and safety. The thalidomide tragedy remains a landmark example of the need for thorough stereochemical evaluation of pharmaceutical compounds [4,5].

Outside the pharmaceutical field, chirality is also highly relevant in the food industry, where it influences the sensory, nutritional, and biological properties of numerous compounds. Differences in enantiomeric composition can affect aroma, flavour, and consumer perception, as exemplified by limonene, whose enantiomers exhibit distinct citrus notes. Likewise, the stereochemical configuration of certain food additives and sweeteners can determine their sensory characteristics [6].

The relevance of chirality extends further to the plant kingdom, where many bioactive molecules possess one or more stereogenic centres. Plants produce a diverse range of secondary metabolites that, although not directly involved in primary metabolism, play essential roles in defence, communication, and ecological interactions [7]. Among these compounds are pigments, terpenes, isoprenoids, and alkaloids, the latter representing one of the largest and most structurally diverse groups of plant-derived bioactive molecules. Owing to their wide range of biological activities and toxicological effects, alkaloids have attracted considerable interest in the pharmaceutical, toxicological, and food fields.

Alkaloids are a diverse group of nitrogen-containing compounds, typically characterised by the presence of one or more nitrogen atoms within a heterocyclic structure. Although they occur in a wide range of organisms, including bacteria, fungi, and animals [8,9], they constitute one of the largest classes of plant secondary metabolites, with more than 21,000 structures described to date [10]. In plants, alkaloids are primarily involved in defence against herbivores, pathogens, and other environmental stressors, although they also contribute to growth, development, and ecological interactions [11,12,13]. Owing to their broad spectrum of biological activities, numerous alkaloids have important pharmaceutical applications, including antispasmodic agents such as atropine, analgesics such as morphine and codeine, and anticancer compounds such as vinblastine [14]. Many alkaloids exert their effects through interactions with neurotransmitter systems and other biological targets, leading to a wide range of pharmacological and toxicological responses. In many cases, these activities are strongly influenced by molecular stereochemistry, as the spatial arrangement of stereogenic centres governs binding affinity and selectivity towards biological receptors. Among the alkaloid families in which chirality plays a particularly important role, tropane alkaloids (TAs) are of special interest owing to the marked influence of stereochemistry on their pharmacological activity and toxicological effects [15].

Given the growing relevance of TAs in pharmaceutical, biological, and food-related fields and the important role of stereochemistry in determining their biological and toxicological properties, reliable methods for enantiomeric separation and determination are increasingly required. Accordingly, this review provides a comprehensive overview of the chiral analysis of TAs, with particular emphasis on liquid chromatographic-based enantiomeric separation strategies, chiral stationary phases (CSPs), and sample preparation approaches applied to pharmaceutical, biological, and food matrices. Particular attention is given to the evolution and application of CSP-based strategies, the performance of these approaches in real matrices, and the preservation of enantiomeric integrity during sample preparation. For this purpose, the literature published between 2010 and 2026 has been critically reviewed to summarise current advances and future challenges in this field.

## 2. Chiral Tropane Alkaloids: Pharmacological and Food Implications

**Figure 1:**
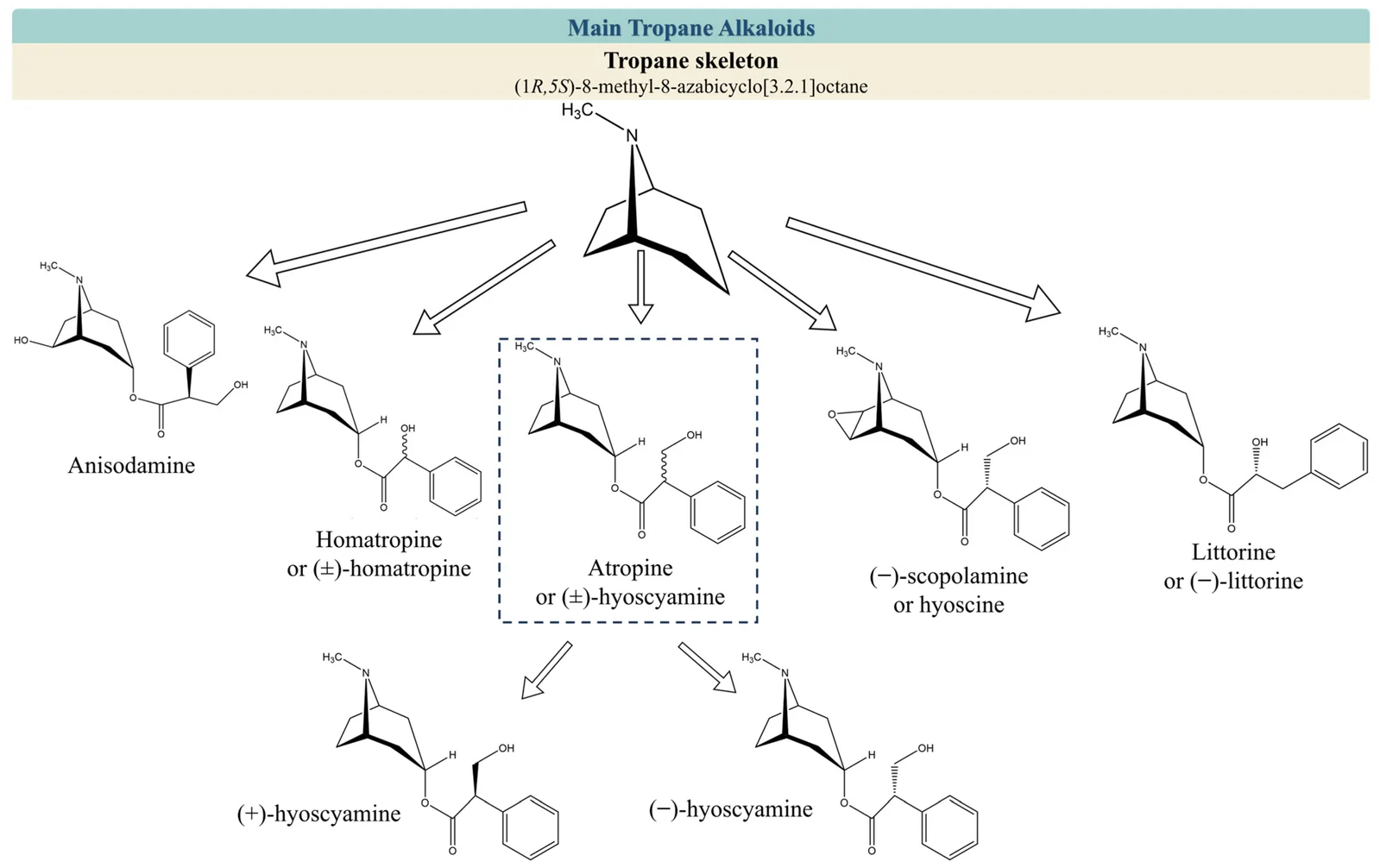
Structure of main tropane alkaloids (TAs) and their chirality.

TAs constitute an important class of plant alkaloids characterised by the presence of a tropane ring, formally designated as (1R,5S)-8-methyl-8-azabicyclo[3.2.1]octane according to IUPAC nomenclature (Figure 1) [16,17]. These compounds are secondary metabolites produced in several plant families, including *Brassicaceae*, *Convolvulaceae*, *Erythroxylaceae*, *Euphorbiaceae*, *Proteaceae*, *Rhizophoraceae*, and *Solanaceae*, with more than 200 identified compounds distributed throughout the plant kingdom [16,18]. Among these families, Solanaceae is of particular relevance owing to the abundance, diversity, and biological activity of its TAs, many of which are formed through the esterification of tropane-derived alcohols and acids [16,19].

Many TAs, particularly those found in *Solanaceae*, possess one or more stereogenic centres and therefore may occur as different stereoisomeric forms. Their stereochemical composition can influence pharmacological activity, toxicity, biosynthesis, and analytical behaviour. Consequently, the characterisation of TA enantiomers is relevant not only to pharmaceutical analysis but also to toxicological assessment and food safety, particularly for TAs occurring as natural contaminants in plant-derived foods [20].

The main chiral TAs considered in the literature reviewed in this work are summarised in Table 1. These compounds differ considerably in their stereochemical characteristics, natural occurrence, pharmacological or toxicological relevance, and analytical challenges. Hyoscyamine enantiomers or atropine (corresponds to the racemic form (±)-hyoscyamine) and scopolamine are particularly relevant because of their occurrence in TA-producing plants and their importance in pharmaceutical applications and food contamination. Other compounds, such as homatropine, anisodamine, and littorine, provide additional examples of the structural and stereochemical diversity of chiral TAs and illustrate different analytical challenges associated with their enantiomeric or stereoisomeric determination.

Among these compounds, atropine and scopolamine are particularly important from a food-safety perspective because of their widespread occurrence in TA-producing plants and their frequent involvement in contamination events. Cereals, legumes, pseudocereals, herbal infusions, and derived food products may become contaminated through the accidental co-harvesting of plants belonging to genera such as Datura, Atropa, and Hyoscyamus [16,20]. Since plant biosynthesis predominantly yields the naturally occurring (−)-hyoscyamine and (−)-scopolamine enantiomers, the determination of their enantiomeric composition may provide information beyond total TA concentration. In particular, deviations from the expected natural enantiomeric composition may indicate changes occurring during processing, storage, or sample preparation, although such changes should be distinguished from other chemical transformations such as hydrolysis.

From an analytical perspective, these differences in stereochemical composition and biological relevance highlight the need for selective methods capable of resolving TA enantiomers or stereoisomers in pharmaceutical, biological, and food matrices. Consequently, the following section focuses on the chromatographic strategies developed for their enantioseparation, with particular emphasis on chiral stationary phases, chromatographic conditions, and detection approaches.

## 3. Strategies for the Enantioseparation of Tropane Alkaloids by Liquid Chromatography

### 3.1. Literature Search Strategy

A focused literature search was conducted in ScienceDirect in January 2026 to identify studies published between 2010 and 2026 addressing the chiral separation or enantioselective determination of TAs. The search combined “tropane alkaloids” with “chiral” or “enantioseparation” and included individual TA names (hyoscyamine, atropine, scopolamine, anisodamine, littorine, and homatropine) combined with “chiral” and related enantioselective terms. The search was restricted to English-language research articles. Records were screened manually by title and abstract, followed by full-text assessment when appropriate. Studies were included if they reported the chiral separation or enantioselective determination of at least one TA and provided sufficient information to identify the analytical technique and its application, with particular emphasis on pharmaceutical products, biological samples, and foodstuffs. Studies were excluded if they did not address TA enantioseparation or enantioselective determination, fell outside the defined publication period, or were not research articles.

**Figure 2:**
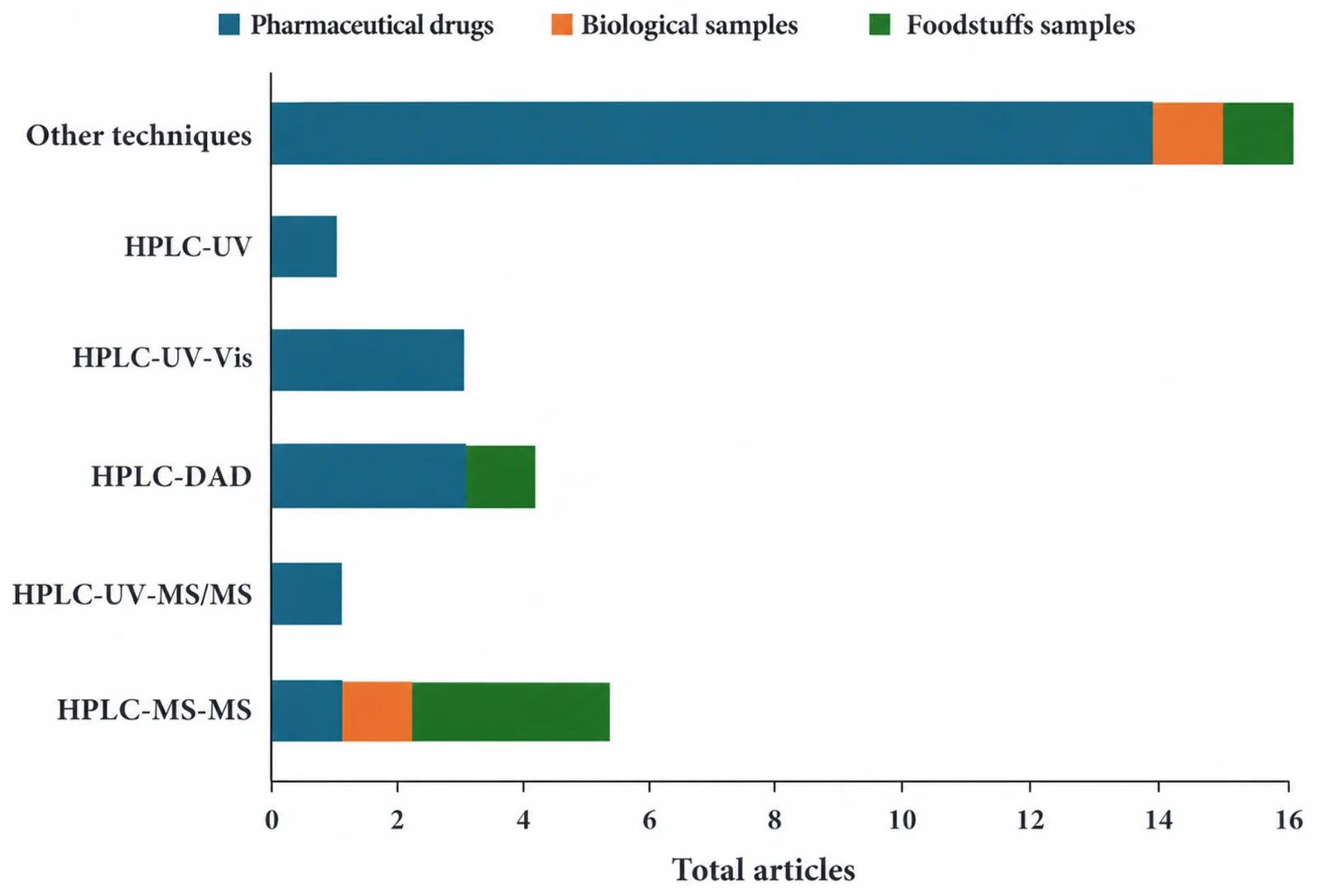
Distribution of studies published between 2010 and 2026 on the chiral determination of tropane alkaloids (TAs), according to analytical platform, HPLC detection system, and sample type. HPLC-based studies are classified according to the detection system used (UV, UV-Vis, DAD, UV-MS/MS, and MS/MS), whereas other analytical techniques include TLC, GC, SFC, and CE. Sample types include pharmaceutical drugs, biological samples, and foodstuffs.

Following screening, 29 unique studies were included in the TA-specific dataset. Thirteen studies employed HPLC-based methods, whereas 16 used other analytical techniques, including thin-layer chromatography (TLC), gas chromatography (GC), capillary electrophoresis (CE), and supercritical fluid chromatography (SFC). HPLC therefore accounted for 44.8% of the identified studies, while the other techniques accounted for 55.2%. The HPLC studies were further classified according to the detection systems employed. As some studies used more than one detection system during method development and final application, the resulting categories are not mutually exclusive. In total, 14 HPLC analytical configurations were identified: HPLC-UV (*n* = 1), HPLC-UV-Vis (*n* = 3), HPLC-DAD (*n* = 4), HPLC-UV-MS/MS (*n* = 1), and HPLC-MS/MS (*n* = 5). The distribution of the identified studies according to analytical platform, detection system, and sample type is presented in Figure 2.

HPLC-based enantioseparation was the most frequently used chromatographic platform in the TA-specific literature; accordingly, the discussion in this section focuses on HPLC methods and their analytical performance. The identified studies were examined in terms of CSP chemistry, chromatographic mode, mobile-phase composition and additives, detection strategy, analytical performance, matrix application, and compatibility with mass spectrometric detection.

Chiral separations can be performed using direct approaches. Direct enantioseparation relies on differential interactions between enantiomers and a chiral selector, incorporated either in the mobile phase or, more commonly, in the stationary phase as a CSP [21,22,23]. The TA-specific methods are included in Table 2. The CSP families represented in these studies include polysaccharide derivatives, cyclodextrins (CD), macrocyclic glycopeptides/antibiotics, proteins, and synthetic or emerging selectors.

Chiral recognition is not determined by CSP identity alone. Depending on the selector and chromatographic conditions, hydrogen bonding, π–π interactions, dipole–dipole interactions, hydrophobic or inclusion interactions, ionic interactions, and steric complementarity may contribute to the formation of transient diastereomeric complexes [24]. Consequently, the same CSP may exhibit markedly different selectivity when the solvent, additives, pH, ionic strength, or temperature is changed. These factors are particularly relevant for basic analytes such as TAs, because mobile-phase composition and additives can affect retention, peak shape, enantioresolution, and compatibility with MS detection.

Table 2 summarises the reported HPLC methods using a common set of analytical descriptors. Parameters not reported in the original studies are indicated as NR rather than inferred. The following subsections discuss the methods according to CSP family and critically compare their chromatographic conditions, chiral recognition characteristics and analytical performance.

**Table 2:** Comparative overview of chiral liquid-chromatographic methods reported for tropane alkaloids between 2010 and 2026, including CSP family, chromatographic mode, mobile phase/additives, detection, resolution, and selected analytical-performance descriptors.

Chiral Stationary Phase (CSP) *	Analytes	Mode/Mobile Phase and Additives	Detector	Rs/α/k′	Time/ LOD–LOQ	Matrix	[Ref.]
Polysaccharide-based CSPs							
*Commercial* polysaccharide-based CSPs: -Cellulose tris(3,5 dimethylphenylcarbamate)/Lux cellulose-1 -Cellulose tris(3-chloro-4-methylphenylcarbamate)/Lux cellulose-2 -Amylose tris(5-chloro-2-methylphenylcarbamate)/ Lux amylose-2 (For UV: 250 × 4.6 mm, 5 µm and for MS/MS: 150 × 2.1 mm, 3 µm)	Atropine, homatropine, and 207 other chiral compounds	Different conditions for the drugs in isocratic elution mode Atropine: MeOH/NH_4_HCO_3_ (20 mM) (60:40 *v*/*v*)—Lux Cellulose-1 Homatropine: MeOH/NH_4_HCO_3_ (20 mM) (80:20 *v*/*v*)—Lux Cellulose-1	UV MS/MS	Enantioseparation of Atropine: 1.5 Homatropine: 4.4 α/k′ NR	NR/NR	Standard solutions	[25]
*Commercial* polysaccharide-based CSP: Amylose tris (3-chlorophenylcarbamate) (250 × 4.6 mm, 5 µm)/Chiralpak ID	Atropine sulfate, homatropine methylbromide, scopolamine hydrobromide and 5 chiral compounds	Isocratic elution mode Atropine sulfate: Hex/EtOH/DEA/FA (60:40:0.1:0.1, *v/v/v/v*) Homatropine methylbromide: Hex/1-propanol/DEA (85:15:0.1, *v*/*v*/*v*)	UV-Vis	Atropine sulfate: 3.98 Homatropine methylbromide: 1.80 Scopolamine hydrobromide: Not separated α/k′ NR	NR/NR	Standard solutions	[26]
*Commercial* polysaccharide-based CSPs: Cellulose tris(4-chloro-3-methylphenylcarbamate) (250 × 4.6 mm, 5 µm)/Daicel ChiralCEL OX-H	Synthetic anisodamine	Isocratic elution mode n-hex-EtOH (90:10, *v*/*v*) or n-hex-*i*-PrOH (85:15, *v*/*v*) containing 0.1% DEA	UV	Rs/α/k′ NR	NR/NR	Standard solutions	[27]
*Commercial* CSPs -Polysaccharide-based CSPs: Amylose tris (5-chloro-2-methylphenylcarbamate) (150 × 4.6 mm, 3 µm)/Chiralpak^®^ AY3 -Macrocyclic antibiotics-based CSPs: Vancomycin covalently bonded to silica (250 × 4.6 mm, 5 µm)/Chirobiotic^®^ V	Atropine	Isocratic elution mode EtOH with 0.1% (*v*/*v*) DEtOHA	MS/MS	Chirobiotic^®^ V: enantioseparation not achieved Chiralpak^®^ AY3: NR	10 min; LOD 0.5 µg/kg; LOQ 1 µg/kg	Standard solutions Solanaceae seeds; Contaminated buckwheat	[28]
*Commercial* polysaccharide-based CSPs: -Amylose tris-(5-chloro-2-methylphenylcarbamate) (150 mm × 4.6 mm, 3 μm)/Chiralpak^®^ AY3 -Amylose tris-(3,5-dimethylphenylcarbamate) (250 mm × 4, 6 mm, 5 μm)/Chiralpak^®^ AD-H -Cellulose tris-(3,5-dimethylphenylcarbamate) (250 mm × 4.6 mm, 5 μm)/Lux^®^ Cellulose-1	Atropine	Isocratic elution mode EtOH with 0.05% DEA	DAD MS/MS	DAD: Rs: 4.30; K_1_: 1.29; K_2_: 1.47 MS: Rs: 1.59	6.5 min; MDL 4.4/4.6 µg/L; MQL 14.8/15.3 µg/L in extract (0.089/0.092 µg/L in infusion)	Standard solutions Baby herbal infusions	[29]
Cyclodextrin-based CSPs							
*Laboratory-made* functionalised β-CD-based CSPs: Mono-6-azido-perphenylcarbamated-β-CD Packed into stainless-steel column (250 mm × 4.6 mm)	Atropine and 30 chiral compounds	Isocratic elution mode MeOH/TEAA buffer (1%, pH 6.5) (50:50, *v*/*v*)	DAD	Rs 2.89; α 1.63; k_1_ 0.74, k_2_ 1.21	NR/NR	Standard solution	[30]
*Laboratory-made* functionalised β-CD-based CSP: Native-β-CD + perphenylcarbamoylated β-CD Packed into stainless-steel column (150 mm × 4.6 mm)	Atropine and 33 chiral compounds	Isocratic elution mode MeOH/H_2_O with 1% TEAA pH 6.25 (30:70, *v*/*v*)	DAD	Rs 1.72; α 1.48; k′ NR	NR/NR	Standard solution	[31]
Macrocyclic antibiotic/glycopeptide CSPs							
*Commercial* macrocyclic antibiotics-based CSPs: Vancomycin (250 × 4.6 mm ID, 5 µm particle size)/Chirobiotic^TM^ V	Tropine, atropine, scopolamine, homatropine, anisodamine, and 2 glycoalkaloids	Isocratic elution mode 10 mM HCO_2_NH_4_ in H_2_O/ACN (90:10, *v*/*v*) and MeOH/ACN (50:50, *v*/*v*) mixed in the ratio 20:80 (*v*/*v*)	MS/MS	No complete enantiomeric resolution; α/k′ NR	NR LOQ 2.2–4.9 ng/g	Standard solutions Food/feed matrices	[32]
*Commercial* CSPs -Macrocyclic antibiotics-based CSPs: Native Vancomycin (200 × 4.6 mm)/Vancoshell column Modified macrocyclic glycopeptide (100 × 4.6 mm)/Nicoshell column -CDs-based CSP: Hydroxypropyl- β-CD (150 × 4.6 mm)/CDShell-RSP column -Cyclofructan-based CSP: Cyclofructan-based (100 × 4.6 mm)/LarihcShell-P column	Atropine, homatropine, and 148 chiral compounds	Isocratic elution mode Nicoshell column: Atropine: MeOH/HCO_2_NH_4_ (100:0.2, *v/w*) Homatropine: MeOH/HCO_2_NH_4_ (100:0.5, *v/w*) ACN-NH_4_HCO_2_ (pH 3.6; 48 mM) (10:90, *v*/*v*) Hep-EtOH-TFA-TEA (90:10:0.3:0.2, *v/v/v/v*)	MS/MS	Atropine Nicoshell column: 1.7 Homatropine Nicoshell column: 3.1 CDShell-RSP column: 2.2 Vancoshell column: 1.5 α/k′ NR	NR/NR	Standard solutions	[33]
Protein-based CSPs							
*Commercial* protein-based CSP: α1-acid glycoprotein (150 mm × 4.0 mm, 5 µm)/Chiral AGP^®^ column	Atropine and zopiclone	Isocratic elution mode hyoscyamine: Sodium phosphate buffer (100 mM) with TEA (42.8 mM) (pH 7)/ACN (90:10 *v*/*v*)	UV-Vis	Hyoscyamine Rs 1.60; zopiclone Rs 2.45; α 1.29/1.47	NR LOD/LOQ 0.156/0.514 µg/mL (hyoscyamine)	Standard solutions	[34]
*Commercial* protein-based CSPs: α1-acid glycoprotein 2 consecutively coupled chiral AGP columns (150 mm × 2.0 mm, 5 µm) and precolumn AGP (10 mm × 2.0 mm, 5 µm)/Chiral-AGP	Atropine	Isocratic elution mode 0.01 M HCO_2_NH_4_, pH 8.0 and 0.01 M HCO_2_NH_4_ in 25%, *v*/*v* ACN, pH 8.0 in 85:15 (*v*/*v*)	MS/MS	Rs/α/k′ NR	50 min; LOD/LOQ NR	Standard solutions Human plasma	[35]
Synthetic and emerging CSPs							
*Commercial* synthetic polymer-based CSP: Poly (N-acryloyl-S-phenylalanine ethyl ester) (250 × 4.0 mm, 5 µm)/ChiraSpher NT column	Atropine and (1RS,2SR)-(±)-ephedrine	Isocratic elution mode MeOH/H_2_O with 0.2% AcOH (60:40, *v*/*v*)	DAD	Atropine: Rs 3.80; α 1.87; k_1_ 2.43 (1RS,2SR)-(±)-ephedrine: Rs 3.37; α 1.46; k_1_ 2.57	NR/NR	Standard solutions Atropine eye drops	[36]
*Laboratory-made* IL-type of CSP: chiral imidazolium, 1-phenylethylamine and 1-(1-napthyl)ethylamine Packed into stainless-steel column (250 mm × 4.6 mm)	Atropine and 18 chiral compounds	Isocratic elution mode 1-phenylethylamine: MP1:ACN/H_2_O (40:60 *v*/*v*) 1-(1-napthyl)ethylamine: MP2: ACN/H_2_O with 0.05% TFA (40:60 *v*/*v*) MP3: ACN/20 mM KH_2_PO_4_ (pH 5.5) (45:55 *v*/*v*)	UV-Vis	Atropine: 1-phenylethylamine: MP1: 0.0 1-(1-napthyl)ethylamine: MP1: 0.0 MP2: 1.05 MP3: 0.69 α/k′ NR	NR/NR	Standard solutions	[37]

* CSP, chiral stationary phase; commercial name and chiral selector are indicated when applicable; laboratory-made or commercial CSPs are specified where relevant. ACN, acetonitrile; AcOH, acetic acid; AGP, α1-acid glycoprotein; CDs, cyclodextrins; DAD, diode-array detector; DEA, diethylamine; DEtOHA, diethanolamine; EtOH, ethanol; FA, formic acid; Hep, heptane; Hex, hexane; ILs, ionic liquids; *i*-PrOH, isopropanol; LOD, limit of detection; LOQ, limit of quantification; MeOH, methanol; MDL, method detection limit; MP, mobile phase; MQL, method quantification limit; MS/MS, tandem mass spectrometry; NR, not reported; Rs, chromatographic resolution; TEA, triethylamine; TEAA, triethylammonium acetate; TFA, trifluoroacetic acid; UV, ultraviolet detector; UV-Vis, ultraviolet-visible detector. The mobile-phase composition and additives are given for the specific analytes and CSPs indicated. Where reported, Rs denotes chromatographic resolution, α the selectivity factor, and k′ the retention factor. LOD, LOQ, MDL and MQL values are reported as provided in the original studies and may refer to different analytical stages or sample matrices. NR indicates that the corresponding parameter was not reported in the source study.

### 3.2. Polysaccharide-Based CSPs

Polysaccharide-derived CSPs are the most extensively represented family in the TA-specific HPLC literature [25,26,27,28,29]. Their broad applicability reflects the ability of cellulose- and amylose-derived selectors to establish multiple stereoselective interactions, but the reported results show that selectivity is highly dependent on the exact selector and chromatographic environment rather than on the polymer class alone [38,39].

Peng et al. [25] demonstrated that polysaccharide phases can operate Reversed-Phase (RP) mode and remain compatible with HPLC-MS/MS. Atropine and homatropine achieved resolution values of 1.5 and 4.4, respectively, on Lux Cellulose-1 under different MeOH-based conditions. Although these results show that both compounds can be separated using the same CSP, the substantially different resolutions highlight the importance of analyte-specific optimisation. Since selectivity factors (α) and retention factors (k′) were not reported for these TA separations, the differences cannot be attributed solely to changes in relative retention.

Under Normal-Phase (NP) conditions, Li et al. [26] obtained an Rs of 3.98 for atropine and 1.80 for homatropine methylbromide using Chiralpak ID, whereas scopolamine was not enantioseparated. For atropine, the mobile phase comprised Hex/EtOH/DEA/FA (60:40:0.1:0.1, *v/v/v/v*), while homatropine required Hex/1-*i*-PrOH/DEA (85:15:0.1, *v*/*v*/*v*). These findings indicate that both the alcohol modifier and the additive system must be adapted to the analyte. The failure to separate scopolamine also demonstrates that successful resolution of one TA does not guarantee equivalent performance for structurally related compounds. Marín-Sáez et al. [28] compared Chirobiotic V with Chiralpak AY3 for the enantioseparation of hyoscyamine and found that the polysaccharide-based AY3 provided the more suitable separation. Wu et al. [27] further demonstrated the applicability of a cellulose-based CSP to the preparative separation of the four stereoisomers of synthetic anisodamine. However, because the latter study focused on preparative isolation rather than quantitative analytical validation, its results are not directly comparable with the analytical performance figures reported in other studies.

**Figure 3:**
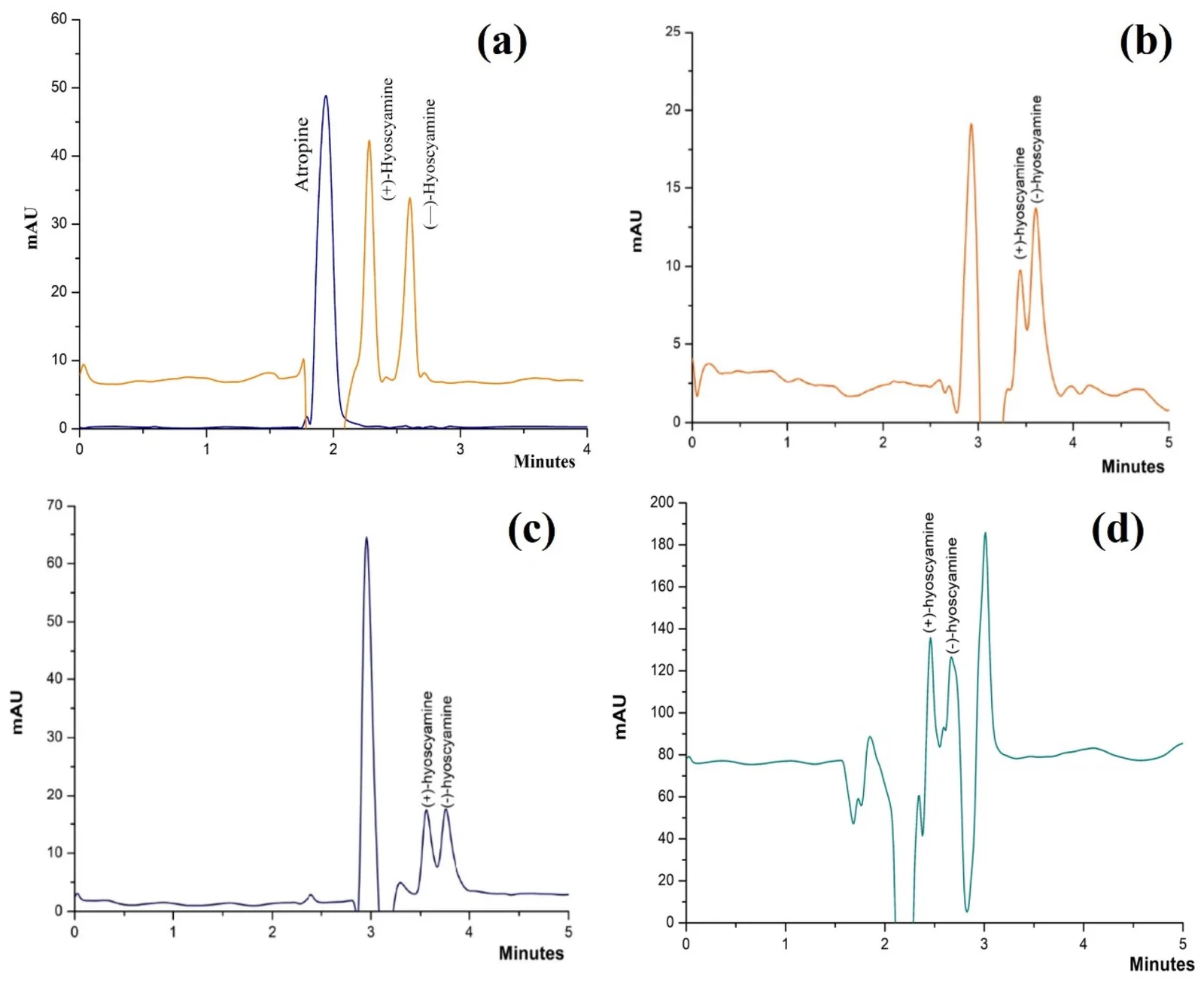
Chiral chromatographic separation of atropine enantiomers by HPLC-DAD in (**a**) Chiralpak^®^ AY-3 with ethanol (blue) or ethanol with 0.1% diethylamine (yellow) as mobile phase. (**b**) Lux^®^ Cellulose-1 with ethanol with 0.1% diethylamine as mobile phase. (**c**) Lux^®^ Cellulose-1 with methanol with 0.1% diethylamine as mobile phase. (**d**) Chiralpak^®^ AY-3 with hexane/ethanol (90/10, *v*/*v*) with 0.1% diethylamine as mobile phase. Flow rate 1 mL/min, room temperature, λ = 230 nm. CSPs are cited with commercial names. Reproduced from reference [29] by the authors (© 2024 The Author(s), published by Elsevier B.V).

More recently, Vera-Baquero et al. [29] conducted a systematic comparison of three polysaccharide-based CSPs, namely Chiralpak^®^ AD-H, Chiralpak^®^ AY-3, and Lux^®^ Cellulose-1, for the enantioseparation of hyoscyamine under NP, RP, and Polar Organic (PO) conditions. Figure 3 illustrates the influence of the CSP and mobile-phase composition during the HPLC-DAD optimisation, showing marked changes in retention, peak shape, and enantiodiscrimination when EtOH, MeOH, Hex/EtOH, and DEA containing mobile phases were used.

The best chromatographic performance was obtained with Chiralpak^®^ AY-3 under PO conditions using EtOH containing 0.1% DEA during the optimisation stage, with an enantioresolution of approximately 4.30. However, the final method was subsequently adapted to HPLC-MS/MS by reducing the DEA concentration to 0.05% to minimise suppression of the ESI(+) response, while retaining adequate enantioseparation (Rs = 1.59) [29]. This example highlights that the condition providing the highest chromatographic resolution is not necessarily the optimal condition for the complete analytical workflow, particularly when MS compatibility and sensitivity are required (Table 2).

Overall, polysaccharide-based CSPs offer the broadest range of demonstrated chromatographic options for TAs, including NP, RP, and PO modes. The evidence supports the use of alcohol modifiers and, where appropriate, amine additives to optimise separation of these basic analytes. Nevertheless, the mobile phase must be tailored to each TA and to the intended detection system, particularly when HPLC-MS/MS is required.

### 3.3. Cyclodextrin-Based CSPs

CD-based CSPs provide a recognition environment that combines host–guest inclusion within the CD cavity with secondary interactions, including hydrogen bonding, dipole–dipole interactions, π–π interactions, and steric complementarity. The relative contribution of these interactions depends on the CD derivative and the mobile phase, which may compete with analyte inclusion or modify the interaction environment [24,40].

**Figure 4:**
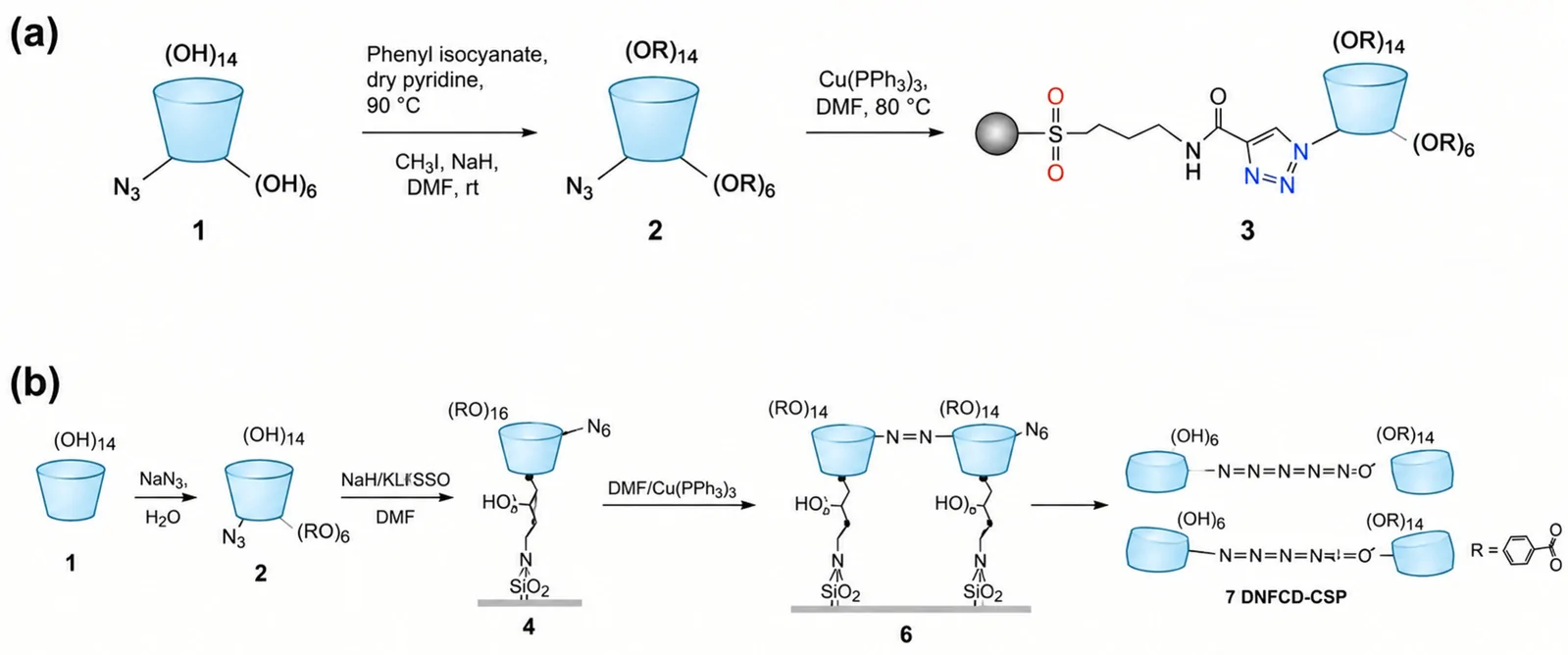
Synthetic routes for the preparation of perphenylcarbamoylated *β*-cyclodextrin-based chiral stationary phases (CSPs). (**a**) Preparation of mono-6-azido-perphenylcarbamated-*β*-CD CSP. (**b**) Preparation of native-perphenylcarbamoylated *β*-CD CSP (DNPCD-CSP). Redrawn, combined, and adapted from [30] and [31], respectively.

The application of these CSPs to TA analysis has mainly focused on functionalised β-CD derivatives. Wang et al. [30] developed a perphenylcarbamoylated β-CD CSP immobilised through click chemistry (Figure 4a). Using MeOH/1% TEAA buffer at pH 6.5 (50:50, *v*/*v*), the column separated atropine with an Rs of 2.89, a selectivity factor (α) of 1.63, and retention factors of k′1 = 0.74 and k′2 = 1.21. These results indicate both measurable enantioselectivity and moderate retention under the reported conditions.

Zhao et al. [31] subsequently developed a bilayer CSP combining native β-CD and perphenylcarbamoylated β-CD (Figure 4b). With MeOH/water containing 1% TEAA at pH 6.25 (30:70, *v*/*v*), atropine achieved an Rs of 1.72 and α of 1.48. The selectivity factor was therefore lower than that reported by Wang et al. [30]. Although the bilayer architecture introduced additional potential recognition sites, it did not provide higher resolution under the reported conditions. This comparison should nevertheless be interpreted cautiously because the CSP structure and mobile-phase composition differed between studies.

Hellinghausen et al. [33] also evaluated a hydroxypropyl-β-CD superficially porous phase (CDShell-RSP), which provided an Rs of 2.2 for homatropine. This result supports the potential of CD-based phases to provide complementary selectivity for particular TAs. However, the available studies do not establish a universally optimal mobile-phase composition for this CSP family, nor do they demonstrate broad validation in real food matrices.

Overall, CD-based CSPs offer a complementary recognition mechanism for TA enantioseparation. The reported results suggest that functionalisation of the CD rim and the proportion of organic modifier can influence selectivity, but greater structural complexity does not necessarily improve resolution. Their performance should therefore be evaluated using resolution and selectivity together rather than retention alone.

### 3.4. Macrocyclic Antibiotic/Glycopeptide CSPs

Macrocyclic glycopeptide or antibiotic CSPs, including vancomycin-based phases, offer multiple potential recognition sites and can combine ionic, hydrogen-bonding, hydrophobic, π–π, and steric interactions [41]. In the TA literature, Jandrić et al. [32] used Chirobiotic V for HPLC-MS/MS analysis of several TAs in food and feed matrices. Although the method achieved low LOQ, complete enantiomeric resolution was not obtained. Its analytical sensitivity should therefore not be interpreted as evidence of enantiomer-specific quantitative capability.

Hellinghausen et al. [33] provided a comparative evaluation of macrocyclic glycopeptide CSPs. NicoShell achieved an Rs of 1.7 for atropine and 3.1 for homatropine, whereas VancoShell achieved an Rs of 1.5 for homatropine. The differences between these results demonstrate that the performance of macrocyclic CSPs depends on the specific selector and analyte. Because selectivity and retention factors were not reported for these TA separations, the observed resolution differences cannot be attributed to relative retention alone. The mobile-phase conditions reported by Hellinghausen et al. [33] also varied according to the column and analyte, illustrating the need for method-specific optimisation. However, the available data do not support a single recommended mobile-phase composition for all macrocyclic CSPs used with TAs. Compared with the best resolutions obtained using some polysaccharide and CD phases, the principal contribution of macrocyclic CSPs in the reviewed literature is their complementary selectivity and applicability to MS-oriented workflows. Their suitability for enantiomer-specific quantification must be established for each method, particularly when complete resolution is not demonstrated.

### 3.5. Protein-Based CSPs

Protein-based CSPs, particularly those containing α1-acid glycoprotein (AGP), provide a chiral three-dimensional binding environment and are commonly operated with aqueous buffered mobile phases. For basic analytes such as TAs, ionic, hydrogen-bonding, hydrophobic, and van der Waals interactions may contribute to retention and stereoselectivity [34,35,42].

Zaazaa et al. [34] reported an Rs of approximately 1.60 for hyoscyamine sulfate on a Chiral AGP column using a mobile phase containing 100 mM sodium phosphate and 42.8 mM TEA at pH 7, combined with ACN (90:10, *v*/*v*). The reported selectivity factors for hyoscyamine and zopiclone were 1.29 and 1.47, respectively. Zopiclone is not a TA but is included here because it was investigated alongside hyoscyamine enantiomers in the original study. These results indicate measurable chiral discrimination, although the hyoscyamine enantiomers resolution was more limited than the highest values reported for some polysaccharide-based CSPs.

John et al. [35] used two AGP columns coupled to HPLC-ESI-MS/MS for enantiomer-specific analysis of hyoscyamine in human plasma. The method used ammonium formate at pH 8 with ACN and required a 50 min chromatographic run. Because a numerical Rs and selectivity factor were not reported in the information available for this review, the separation cannot be quantitatively compared with methods for which these parameters are available. Nevertheless, the study illustrates the use of protein-based CSPs in a biological matrix with MS/MS detection.

The reviewed evidence suggests that AGP phases can provide useful enantioselectivity under buffered conditions and can be integrated into HPLC-MS/MS workflows. However, the available TA-specific studies are too limited to establish a generally optimal pH or mobile-phase composition or to conclude that protein-based CSPs outperform other families. Their practical value should be assessed in relation to the target analyte, the required sensitivity, and the overall analytical time.

### 3.6. Synthetic and Emerging CSPs

Synthetic and emerging CSPs provide opportunities to tailor selector chemistry and explore recognition mechanisms that differ from those of established polysaccharide, CD and protein phases. However, the TA-specific evidence remains limited and is dominated by standard-solution studies.

Kumar et al. [36] obtained an Rs of 3.80, a selectivity factor of 1.87 and a retention factor k′1 of 2.43 for atropine on the polyacrylamide-based ChiraSpher NT column using MeOH/water containing 0.2% AA (60:40, *v*/*v*). These results demonstrate useful enantiodiscrimination under the reported conditions. However, the study also showed that prolonged stopped-flow and low-flow conditions could promote hyoscyamine enantiomerisation. Consequently, the chromatographic conditions must be evaluated not only for resolution and retention but also for their potential effect on the original enantiomeric ratio.

Wang et al. [37] investigated chiral imidazolium CSPs derived from 1-phenylethylamine and 1-(1-naphthyl)ethylamine. Atropine was not resolved on the first selector (Rs = 0). On the naphthyl-derived selector, the mobile phase containing ACN and water with 0.05% TFA (40:60, *v*/*v*) produced an Rs of 1.05, whereas the ACN/phosphate-buffer system (45:55, *v*/*v*; pH 5.5) yielded an Rs of 0.69. Under the TFA-containing condition, the reported selectivity factor was 2.87, and k′1 was 0.17. This combination illustrates that a relatively high selectivity factor does not necessarily produce baseline resolution when retention is limited. The lower resolution obtained with phosphate buffer further demonstrates the influence of mobile-phase chemistry on chiral recognition.

Together, these studies show that synthetic CSPs can provide useful or complementary selectivity, but their performance depends on both selector structure and mobile-phase composition. The current evidence does not support a general recommendation for a single synthetic selector or mobile-phase additive for TAs. Further work should establish whether these phases can deliver robust enantioseparation, suitable retention and validated performance in real matrices.

### 3.7. Cross-Family Comparison: Chromatographic Performance Versus Analytical Utility

The cross-family comparison shows that useful enantioseparation of TAs can be achieved using several CSP families, but the reported resolution values should not be treated as a direct ranking of stationary phases. For example, atropine achieved Rs values of 3.98 on Chiralpak ID [26], 3.80 on ChiraSpher NT [36] and 2.89 on the β-CD-based CSP reported by Wang et al. [30]. However, these results were obtained under different chromatographic conditions and with different experimental configurations. They therefore demonstrate that several selector chemistries can be effective, rather than establishing a universal order of CSP performance.

The selectivity factor and retention factor provide complementary information. The results reported by Wang et al. [30] and Zhao et al. [31] show that the CD-based phase with the higher α also achieved higher resolution under the conditions tested, whereas the retention factors reported by Wang et al. provide additional information on analyte retention. Conversely, the imidazolium phase studied by Wang et al. [30] yielded a selectivity factor of 2.87 but only an Rs of 1.05, with a low reported retention factor. Thus, α should be interpreted alongside Rs and k′ rather than used alone to judge method suitability. Where these parameters were not reported, cross-study interpretation must remain qualitative.

The most useful mobile-phase strategy also depends on the CSP family and the analytical objective. MeOH-based RP conditions with volatile ammonium bicarbonate were successfully used with polysaccharide CSPs and MS/MS detection [25]. In NP systems, alcohol modifiers and amine additives such as DEA were used to optimise the separation of basic TAs [26]. For β-CD CSPs, MeOH/water systems containing TEAA enabled atropine enantioseparation [30,31]. The available evidence does not establish a single preferred mobile phase across these families; rather, it shows that solvent identity, organic fraction, buffer and additive concentration must be optimised for the specific selector–analyte combination.

For basic TAs, amine additives may improve peak shape and chromatographic performance, but their use must be balanced against detector compatibility. The study by Vera-Baquero et al. [29] illustrates this trade-off: the DEA concentration was reduced during transfer from DAD optimisation to the final HPLC-MS/MS method to limit ESI(+) suppression. The implications for detector selection and method transfer are discussed in Section 3.8.

Finally, the most useful method is not necessarily the one with the highest Rs. Its suitability also depends on retention, analysis time, sensitivity, matrix effects, recovery, detector compatibility and preservation of enantiomeric integrity. In the current literature, particular value should be assigned to methods that demonstrate sufficient enantiodiscrimination together with validated performance in real biological or food matrices. Future studies would benefit from reporting Rs, α and k′ alongside validation parameters, thereby enabling more meaningful comparisons between CSP families and analytical applications.

### 3.8. Detection Systems and MS Compatibility

UV and DAD remain useful for pharmaceutical and method-development studies because they are robust and widely available. Most early and screening-oriented studies employed UV or DAD detection, particularly when working with standard solutions or pharmaceutical formulations, where analyte concentrations are relatively high and extensive sample clean-up is generally unnecessary [25,26,27,30,31,36,37]. These detectors also facilitate rapid evaluation of CSP selectivity during method development. However, HPLC-MS/MS provides substantially greater sensitivity and selectivity for biological and food matrices [28,29,32,33,35]. This advantage becomes particularly relevant when TAs are present at trace concentrations or when complex matrices generate substantial chromatographic background. The studies by Jandrić et al. [32], John et al. [35], Marín-Sáez et al. [28], and Vera-Baquero et al. [29] illustrate the value of MS/MS-based detection for applications requiring enhanced sensitivity and/or analysis of complex biological or food matrices.

The choice of detector, however, cannot be considered independently of the chromatographic conditions. For basic TAs, amine additives such as DEA may improve peak shape and, in some cases, enantioresolution by modifying interactions between the analyte and residual silanol groups. Nevertheless, DEA can strongly suppress electrospray ionisation in positive mode. Peng et al. [25] showed that even low concentrations of DEA could markedly reduce analyte response in ESI(+)-MS/MS, highlighting the incompatibility that may arise between conditions optimised for chromatographic resolution and those required for sensitive MS detection.

This trade-off is particularly evident in the study of Vera-Baquero et al. [29]. During HPLC-DAD optimisation, the use of 0.1% DEA provided improved peak shape and an optimised resolution of approximately Rs = 4.30 (Figure 3). When the method was transferred to HPLC-MS/MS, the DEA concentration was reduced to 0.05% to minimise ion-suppression effects. Under the final conditions, Rs decreased to 1.59, but the separation remained suitable for enantiomer-specific determination while providing the sensitivity required for analysis of herbal infusions. This example demonstrates that the chromatographic condition providing the highest Rs is not necessarily the most appropriate for the overall analytical objective. In HPLC-MS/MS methods, chromatographic resolution must therefore be balanced against ionisation efficiency, sensitivity, and matrix effects.

The reviewed studies also show that MS-compatible mobile phases can be used without necessarily compromising chiral discrimination. Peng et al. [25] demonstrated enantioseparation of atropine and homatropine using RP polysaccharide CSPs and volatile ammonium bicarbonate-containing mobile phases, with HPLC-MS/MS detection. Similarly, Jandrić et al. [32] employed volatile ammonium formate-based conditions with a Chirobiotic V column and triple-quadrupole MS/MS for the analysis of TAs in food and feed matrices. These examples indicate that the use of volatile buffers and appropriate organic modifiers can provide a practical compromise between chromatographic performance and MS compatibility.

## 4. Strategies for the Extraction and Sample Preparation of Tropane Alkaloids

Sample preparation is a critical step in analytical workflows because it directly influences the accuracy, sensitivity, and reliability of the results obtained [43,44]. However, among the chiral LC methodologies discussed in the previous section (Table 2), only six included a sample preparation procedure prior to analysis (Table 3). These correspond to studies applied to real pharmaceutical, biological, or food samples, whereas most of the reported enantioselective methods were developed and evaluated using standard solutions and therefore did not require specific extraction or clean-up steps.

The need for sample treatment arises from the complexity of real matrices, which often contain endogenous compounds, excipients, or co-extracted substances that may interfere with chromatographic determination. Consequently, extraction, purification, and, in some cases, preconcentration procedures are required to isolate the target analytes, reduce matrix effects, and achieve the sensitivity necessary for their reliable quantification [45]. These steps are particularly important prior to chromatographic analysis, where efficient analyte recovery, removal of matrix interferences, and reproducible analytical performance are essential [43,44].

The selection of an appropriate sample preparation strategy depends on both the characteristics of the matrix and the physicochemical properties of the analytes. TAs are weak bases and exhibit high water solubility under acidic conditions, a property that has been widely exploited for their selective extraction from complex matrices using acidified aqueous media [44,46]. Such an approach facilitates the separation of TAs from more lipophilic matrix components and may simplify subsequent purification and chromatographic analysis. In addition to analyte solubility, properties such as pKa, lipophilicity, and partition coefficients should also be considered when optimising extraction conditions [46].

As summarised in Table 3, the sample preparation approaches reported for chiral TA analysis were mainly focused on analyte extraction, matrix clean-up, and, when required, concentration enhancement prior to enantioselective determination. The methodologies employed in each study are discussed below. Overall, sample preparation requirements vary substantially according to the nature of the matrix. Pharmaceutical formulations generally involve relatively simple pretreatment procedures, such as dissolution or dilution, owing to their controlled composition and the comparatively high concentrations of the target analytes. In contrast, biological and food matrices are inherently more complex and frequently contain TAs at trace levels, requiring more elaborate extraction, purification, and, in some cases, preconcentration steps to ensure accurate and reliable enantiomeric determination. The following discussion emphasises the balance between extraction efficiency, matrix clean-up, sensitivity, and stereochemical integrity.

### 4.1. Preservation of Enantiomeric Integrity and Racemisation During Sample Preparation

For chiral TA analysis, sample preparation is part of the stereochemical measurement rather than a purely preparative step. Enantiomerisation of a naturally enriched sample can generate the opposite enantiomer and thereby produce a false change in the native profile. Conversely, hydrolysis or dehydration can decrease the concentration of the parent TA enantiomer without producing the opposite enantiomer. These processes must therefore be distinguished experimentally rather than inferred from a decrease in parent concentration alone.

Marín-Sáez et al. [28] explicitly evaluated pH and temperature effects on hyoscyamine. Under selected extraction conditions the procedure avoided significant racemisation, whereas strongly alkaline conditions (pH 9) combined with elevated temperature promoted enantiomerisation. At 80 °C, the initial (−)-hyoscyamine remained approximately 98.5% with 1.5% of the (+)-enantiomer under one tested condition, while prolonged exposure under alkaline conditions drove the system progressively towards a racemic composition. The authors also distinguished inversion from hydrolysis to tropic acid/tropane products and dehydration to apo derivatives.

Kumar et al. [36] provided complementary evidence that chromatographic conditions themselves can affect enantiomeric integrity: prolonged stopped-flow and low-flow conditions promoted measurable enantiomerisation of hyoscyamine, whereas flow rates of at least 1 mL/min minimised the effect. Vera-Baquero et al. [29] further demonstrated that Solid-Phase Extraction (SPE) can introduce enantiomerisation even when the chromatographic separation itself is stable. A racemic control remained approximately 50:50 after SPE at pH 2.5, but individual (−)-hyoscyamine standards showed conversion during the basic ammonia-elution step; mean (−)/(+) ratios of 92.5/7.5 and 80.5/19.5 were observed in two infusion matrices, and correction factors were applied to the final quantification.

These studies indicate practical controls for preserving enantiomeric integrity: minimise exposure to strongly alkaline conditions, elevated temperature, and prolonged residence times; avoid unnecessary evaporation/reconstitution when it can be replaced by direct dilution; evaluate storage stability; and test individual-enantiomer controls through the complete extraction and chromatographic workflow. In addition, enzymatic hydrolysis used intentionally for stereoselective analysis, as in the John et al. method [35], should not be classified as racemisation because it selectively removes one enantiomer rather than interconverting it.

In addition to preserving enantiomeric integrity, sample preparation must be adapted to the characteristics of each matrix and the analytical requirements of the method. The following section reviews the approaches reported for pharmaceutical formulations, biological samples, and food matrices, highlighting the main extraction and clean-up procedures and their implications for chromatographic analysis.

### 4.2. Sample Preparation Across Pharmaceutical, Biological, and Food Matrices

Among the solvents employed in pharmaceutical formulations, MeOH plays a prominent role owing to its excellent solubilisation capacity and compatibility with HPLC-based techniques. Kumar et al. [36] prepared atropine eye drop solution by dissolving the lyophilised sample in MeOH followed by dilution with a MeOH/water mixture (60:40, *v*/*v*) before HPLC-DAD analysis with good recoveries.

Similarly, Zaazaa et al. [34] employed straightforward sample preparation procedures for different pharmaceutical formulations. Commercial Isopto-atropine ophthalmic solutions were directly diluted with the mobile phase, consisting in 100 mM sodium phosphate buffer with 42.8 mM TEA and ACN (90:10, *v*/*v*) at pH 7, prior to HPLC-UV analysis.

In the case of tablet formulations, samples were first dissolved in ACN and subsequently diluted with the mobile phase before chromatographic analysis with recovery values of 98.83–101.51%. These studies illustrate that the chiral analysis of pharmaceutical formulations can often be performed with minimal sample manipulation, reducing the risk of analyte loss or enantiomerisation while simplifying the overall analytical workflow.

In contrast to pharmaceutical formulations, John et al. [35] addressed the determination of hyoscyamine stereoisomers in human plasma using an enantioselective sample preparation strategy based on two parallel procedures. In Procedure A, plasma samples were incubated with human serum lacking atropinesterase to determine total hyoscyamine concentrations, whereas in Procedure B, samples were incubated with rabbit serum containing atropinesterase, which selectively hydrolysed (−)-hyoscyamine, allowing the determination of the remaining (+)-hyoscyamine. Following incubation at 37 °C, both procedures included protein precipitation with ACN and dilution prior to HPLC-MS/MS analysis, providing satisfactory recoveries ranging from 93 to 101% (Table 3). Compared with the simple dissolution and dilution steps typically employed for pharmaceutical formulations, this approach required substantially greater sample manipulation owing to the complexity of the biological matrix and the need to obtain enantiomer-specific information. Nevertheless, it enabled reliable determination of individual hyoscyamine enantiomers in human plasma while minimising matrix-related interferences.

In food matrices, sample preparation plays a critical role in achieving the sensitivity and selectivity required for the determination of TAs at trace levels. Routine HPLC-MS/MS analysis generally requires sufficiently clean extracts to minimise matrix effects, prevent instrument contamination, and preserve analytical sensitivity. Selective extraction dissolves analytes in aqueous or organic solutions, followed by purification and preconcentration, which are essential to meet regulations and detect trace levels [47]. Particular attention must also be paid to extraction conditions, as the natural enantiomeric composition of TAs may be altered by racemisation during sample preparation. Since food contamination is expected to be dominated by the naturally occurring (−)-hyoscyamine produced by TA-containing plants, any modification of the native enantiomeric ratio may compromise the reliability of enantiomer-specific determinations. Previous studies have shown that, under acidic conditions (pH 3), only limited hydrolysis of hyoscyamine occurs, whereas alkaline media and elevated temperatures promote racemisation, leading to the formation of (+)-hyoscyamine and, ultimately, atropine as a racemic mixture [16,46,48]. Consequently, extraction procedures must not only provide efficient analyte recovery but also preserve the original stereochemical composition of the sample. For this reason, optimisation of sample preparation requires careful evaluation of potential enantiomerisation phenomena through suitable chiral analytical methods [49]. To meet these requirements, the methodologies reported to date (Table 3) have combined extraction and clean-up strategies based on solid-phase extraction (SPE), dispersive solid-phase extraction (dSPE), matrix solid-phase dispersion (MSPD), and QuEChERS procedures, depending on the complexity of the matrix and the analytical objectives pursued.

Jandric et al. [32] developed a sample preparation procedure for the determination of tropine, atropine, scopolamine, homatropine, anisodamine, and two glycoalkaloids in a wide variety of food and feed matrices, including wheat, rye, maize, mixed grain flour, maize flour, biscuits, crackers, and animal feed. Given the diversity of the analyzed matrices, different extraction and clean-up strategies were employed according to sample composition (Table 3). For cereal and grain-based samples, the authors applied a dSPE approach. In this technique, the sorbent material is directly dispersed into the sample extract, maximising contact between matrix components and the sorbent, after which the purified extract is recovered by centrifugation. dSPE stands out as a rapid and efficient method for sample cleanup and extraction, which has gained widespread acceptance in sample pretreatment due to its efficacy and simplicity [50]. Extraction was performed using 0.5% FA in ACN/water (75:25, *v*/*v*), whereas a mixture of magnesium sulfate, sodium chloride, and sodium citrate was employed to promote phase partitioning, improve analyte transfer into the extraction solvent, and regulate the extraction medium [32]. For high-fat matrices, the authors employed MSPD, a technique in which the sample is mechanically blended with a solid sorbent, prior to analyte elution. This approach combines extraction and clean-up in a single step and is particularly useful for removing co-extracted lipophilic compounds that may interfere with chromatographic analysis. In the present study, C18 was used to retain non-polar matrix components and improve extract cleanliness before analysis [32,50].

Following both dSPE and MSPD procedures, the extracts were centrifuged, and an aliquot was evaporated to dryness under a nitrogen stream. The residue was then reconstituted in MeOH/ACN (50:50, *v*/*v*), sonicated, and filtered prior to HPLC-MS/MS analysis. The overall methodology provided satisfactory extraction efficiency, with recoveries ranging from 61 to 111% for the target alkaloids [32]. These approaches illustrate how matrix composition strongly influences the selection of extraction and clean-up strategies in food analysis, requiring more extensive sample preparation than that typically employed for pharmaceutical formulations.

Marín-Sáez et al. [28] employed a modified QuEChERS procedure for the extraction of (+)- and (−)-hyoscyamine from *Datura stramonium* seeds, *Brugmansia arborea* seeds, and contaminated buckwheat (*Fagopyrum esculentum*) samples. QuEChERS, an acronym for quick, easy, cheap, effective, rugged, and safe, is widely used in food analysis because it combines efficient extraction with a simple clean-up procedure while minimising sample handling [51]. In this study, the samples were first ground into a fine powder and extracted with water and ACN containing 1% AcOH. The extract was subsequently purified by dispersive solid-phase extraction using primary secondary amine (PSA) and graphitised carbon black (GCB) as sorbents. PSA is commonly employed to remove polar matrix components such as organic acids, sugars, and pigments, whereas GCB effectively eliminates pigments and other planar interferences, resulting in cleaner extracts suitable for HPLC-MS/MS analysis. Following clean-up, the extract was diluted with EtOH (50:50, *v*/*v*) prior to chromatographic determination (Table 3).

A particularly relevant aspect of this methodology was that the extraction and clean-up conditions preserved the native enantiomeric composition of the samples, making the procedure suitable for the enantiomer-specific determination of TAs in food matrices [28]. This feature is especially important in chiral analysis, where sample preparation must minimise potential changes in enantiomeric composition that could compromise the reliability of the results.

In contrast to dSPE, MSPD, and QuEChERS approaches, SPE remains one of the most widely employed techniques for the extraction, clean-up, and preconcentration of TAs in food samples. The technique typically involves conditioning, sample loading, washing, and elution steps, with extraction efficiency depending on the characteristics of the sorbent, the extraction solvents, and the interactions established between the analytes and the solid phase [45,52]. Because TAs are basic alkaloids containing a tertiary amine group, strong cation-exchange (SCX) sorbents have been extensively employed for their selective extraction, allowing efficient retention of protonated analytes while matrix interferences are removed during the washing step [53]. Consequently, mixed-mode polymeric sorbents such as Oasis MCX^®^ and Strata-XC^®^ have become common choices for the extraction of TAs from food samples [52].

In recent years, particular attention has been devoted to the development of new sorbent materials capable of improving extraction performance while reducing sorbent and solvent consumption in accordance with Green Analytical Chemistry (GAC) principles. Among these materials, functionalised mesoporous silicas have attracted considerable interest owing to their high surface area, controlled pore structure, and versatility for chemical modification [50,54]. Following this approach, Vera-Baquero et al. [29] employed a sulfonic acid-functionalised hexagonal mesoporous silica synthesised by co-condensation (HMS-SO_3_-(co)) as a SCX sorbent for the chiral determination of hyoscyamine enantiomers in baby herbal infusions. Prior to SPE, samples were subjected to solid-liquid extraction (SLE) using FA-acidified water, followed by centrifugation and filtration (Table 3). Subsequently, a micro-SPE procedure employing only 50 mg of sorbent was applied, achieving a preconcentration factor of 167 [29]. This miniaturised approach illustrates the potential of advanced mesoporous materials to simultaneously enhance extraction efficiency, reduce solvent and sorbent consumption, and improve the sustainability of analytical methodologies. Following elution with 0.5% ammonia in MeOH, the extracts were directly subjected to chiral HPLC-MS/MS analysis. The proposed procedure provided satisfactory extraction efficiency, with recoveries ranging from 94 to 99% (Table 3). Furthermore, only limited enantiomerisation was observed during sample preparation, indicating that the extraction conditions largely preserved the original enantiomeric composition of the samples. The influence of these conditions on hyoscyamine racemisation is discussed in detail in the following section [29].

## 5. Applications of Chiral Analytical Methodologies for Tropane Alka-Loids: Key Findings from Pharmaceutical, Biological, and Food Matrices

The practical applications of the developed chiral analytical methodologies for TAs in pharmaceutical, biological, and food matrices are reviewed. Relevant studies and their principal findings are summarised in Table 3. Notably, among the liquid chromatographic methods developed for the chiral analysis of TAs and summarised in Table 2, only two have been applied to pharmaceutical samples, one to biological samples, and three to food matrices, highlighting the limited number of studies addressing real-sample analysis (Table 3).

### 5.1. Pharmaceutical and Biological Samples

As discussed in the Introduction and Section 2, several TAs, including atropine, scopolamine, homatropine, and anisodamine, are widely used as therapeutic agents, and their pharmacological and toxicological properties may be strongly influenced by stereochemistry. Since differences in biological activity, efficacy, metabolism, and safety can exist between enantiomers, the determination of enantiomeric composition is of particular importance in pharmaceutical analysis and drug quality control. Likewise, enantioselective analytical methods are valuable tools in biological studies, where the investigation of the stereoselective disposition, metabolism, and elimination of chiral compounds contributes to a better understanding of their pharmacokinetic behaviour. However, despite the pharmaceutical relevance of many TAs and the well-recognised influence of stereochemistry on their biological activity, only a limited number of studies have applied the developed chiral analytical methodologies to real pharmaceutical and biological samples. The available application studies and their main findings are summarised in Table 3 and discussed below.

Kumar et al. [36] investigated the enantiomerisation of atropine and its semipreparative enantioseparation, together with (1RS,2SR)-(±)-ephedrine, using a polyacrylamide-based CSP and HPLC-DAD detection (Table 2). The study demonstrated that the enantiomerisation rates of (−)- and (+)-hyoscyamine differ, although not sufficiently to generate a non-racemic mixture under the investigated conditions. Furthermore, the isolated fractions were shown to be free from enantiomerisation products, confirming the suitability of the semipreparative separation procedure. The method was successfully applied to the determination of the enantiomeric composition of atropine ophthalmic formulations and enabled reliable chromatographic analysis without artefacts associated with enantiomerisation processes.

Similarly, Zaazaa et al. [34] developed an HPLC-UV method for the direct enantioseparation of hyoscyamine sulfate enantiomers using a protein-based CSP (Table 2). The method was intended for the determination of the eutomer and the evaluation of enantiomeric composition in racemic pharmaceutical formulations. Its applicability was demonstrated through the analysis of a commercial Isopto-atropine ophthalmic solution. Excellent analytical performance was achieved, including high selectivity, baseline enantiomeric resolution, and low LOD and LOQ of 0.156 and 0.514 μg/mL, respectively (Table 3). In addition, thermodynamic studies revealed that chiral recognition was governed by both enthalpic and entropic contributions, providing further insight into the separation mechanism. The high resolution obtained also enabled the reliable detection of potential enantiomeric impurities, highlighting the usefulness of this approach for pharmaceutical quality control [34]. Taken together, these studies demonstrate that chiral chromatographic methodologies can be effectively applied to real pharmaceutical formulations, providing accurate determination of enantiomeric composition while supporting the detection of enantiomeric impurities and the quality control of atropine-containing products.

Beyond pharmaceutical preparations, enantioselective methodologies have also been applied to biological samples, where the determination of TAs is particularly challenging due to their low concentrations and the complexity of biological matrices. In such cases, highly selective analytical strategies are required for pharmacokinetic, clinical, and toxicological investigations. In this context, John et al. [35] developed an enantioselective HPLC-ESI-MS/MS assay for the determination of hyoscyamine stereoisomers in plasma. The method was based on the stereospecific hydrolysis of (−)-hyoscyamine by atropinesterase present in rabbit serum, while (+)-hyoscyamine remained unaffected. Plasma samples were incubated separately with human serum and rabbit serum prior to analysis, allowing determination of total hyoscyamine and residual (+)-hyoscyamine concentrations, respectively. The concentration of (−)-hyoscyamine was then calculated by difference. This approach enabled selective, accurate, and reproducible quantification of individual enantiomers, yielding accuracy values between 93 and 101% and RSD values of 2–9% (Table 3).

The applicability of the method was demonstrated through the analysis of human plasma samples obtained from a patient suffering organophosphorus pesticide poisoning and treated with atropine. Potential antidotal therapies commonly used in such intoxications, including carbamates and oximes, as well as thiono-pesticides, did not interfere with the assay. Although some oxon pesticides inhibited the stereospecific hydrolysis of (−)-hyoscyamine, the authors incorporated a control experiment to detect such interferences and avoid erroneous quantification. Furthermore, the results obtained with the enzymatic assay were validated by comparison with an independent chiral HPLC-ESI-MS/MS method employing a protein-based CSP (Table 2), confirming the reliability of the proposed approach [35]. This study demonstrates the potential of enantioselective methodologies for the determination of TAs in biological matrices and highlights their usefulness for pharmacokinetic and toxicological investigations involving atropine administration and poisoning cases.

### 5.2. Food Samples

The occurrence of TAs in food has become a major concern for food safety authorities due to the toxicological risks associated with their consumption and the increasing number of contamination incidents reported in plant-derived food products [52]. Food contamination is primarily attributed to the accidental co-harvesting of seeds, leaves, and other plant fragments from TA-producing weeds, particularly species belonging to the Solanaceae family, together with food crops [13]. As a result, TAs have been detected in a variety of foods, including cereals, cereal-based products, herbal infusions, and foods intended for infants and young children [52]. In response to these concerns, the European Food Safety Authority (EFSA) established in 2013 an acute reference dose (ARfD) of 0.016 μg/kg body weight, expressed as the sum of (−)-hyoscyamine and (−)-scopolamine, and recommended the generation of additional occurrence data and toxicological information to improve risk assessment [16]. EFSA also highlighted that plant biosynthesis predominantly produces the naturally occurring (−)-enantiomers of hyoscyamine and scopolamine, emphasising the potential relevance of enantiomeric composition in food safety studies.

Following these recommendations, the European Union (EU) established maximum levels for atropine (racemic mixture or hiosciamine enantiomers) and scopolamine in specific food categories. Under Regulation (EU) 2023/915, maximum levels of 1 μg/kg apply separately to atropine and scopolamine in specified infant foods and processed cereal-based foods for infants and young children containing millet, sorghum, buckwheat, maize, or their derived products [55]. A different maximum level applies to liquid herbal infusions: 0.20 μg/L. These limits refer to distinct regulatory product categories and must not be used interchangeably when assessing compliance. In particular, the limit for liquid herbal infusions should not be described as the general maximum level for all foods intended for infants and young children [55]. To support compliance with these regulations, analytical methodologies must achieve sufficient sensitivity, selectivity, and reliability to detect and quantify these compounds at the low concentration levels established by current legislation. Accordingly, the European Commission (EC) recommends highly sensitive chromatographic methods, mainly HPLC-MS and, alternatively, GC-MS, capable of quantifying these compounds at trace levels in complex food matrices [20]. The recommended limits of quantification are below 5 μg/kg for agricultural products, ingredients, food supplements, and infusions, below 2 μg/kg for finished products, and below 1 μg/kg for baby food [20]. These analytical performance targets should be distinguished from the legally established maximum levels for individual food categories. The EC further recognises that atropine corresponds to the racemic form of hyoscyamine and has emphasised the value of developing analytical methodologies capable of providing enantiomer-specific information whenever feasible. Such information may be particularly useful for evaluating the occurrence of racemisation processes and for improving the toxicological assessment of contaminated food products.

Despite the growing regulatory interest in TAs, studies addressing their enantioselective determination in food remain scarce. EFSA has therefore highlighted the need for new chiral analytical methodologies, supported by interlaboratory validation studies and appropriate reference materials, to enable reliable enantiomer-specific analysis of these contaminants in food matrices [16]. This need is particularly relevant because sample preparation procedures, including extraction and cleanup steps, may promote partial racemisation of chiral TAs, potentially altering their natural enantiomeric composition, as discussed in Section 4. Moreover, since (−)-hyoscyamine is considered the more pharmacologically active and toxic enantiomer, racemisation towards the less active (+)-form may modify the toxicological significance of a contaminated sample.

Although numerous analytical methods have been developed for the determination of TAs in food, most rely on achiral chromatographic approaches, as recently reviewed by González-Gómez et al. [52]. In contrast, the application of chiral analytical methodologies to food matrices remains scarce. Despite the considerable number of HPLC-based methods reported for the enantioseparation of TAs (Table 2), only three studies have applied these methodologies to the determination of individual enantiomers in real food samples. This limited number of applications highlights the need to further expand the use of chiral analytical methods in food safety studies. The available studies are summarised in Table 3 and discussed below.

Jandric et al. [32] investigated the determination of several TAs, including atropine, scopolamine, homatropine, anisodamine, and tropine, in a variety of grain and seed matrices such as wheat, rye, maize, soybean, and flaxseed. The authors developed an HPLC-MS/MS method using a macrocyclic antibiotic-based CSP (Chirobiotic V^®^) operated under isocratic conditions (Table 2). Although baseline enantioseparation was not achieved, and, therefore, no enantiomer-specific information could be obtained, the method enabled the selective determination of the target alkaloids with satisfactory analytical performance including low limits of quantification (2.2–4.9 ng/g) (Table 3). Application to real samples revealed the presence of atropine and scopolamine in pig and poultry feed, whereas these alkaloids were not detected in products intended for human consumption, such as wheat, rye, maize, and biscuits. Despite the lack of enantiomeric resolution, this work demonstrated the applicability of chromatographic methodologies employing CSP to the determination of TAs in complex food and feed matrices and highlighted the analytical challenges associated with their enantioseparation [32].

Marín-Sáez et al. [28] subsequently developed an HPLC-MS/MS method using a polysaccharide-based CSP (Chiralpak^®^ AY-3) in PO mode for the enantioselective determination of hyoscyamine (Table 2) in *Datura stramonium* seeds, *Brugmansia* seeds, and contaminated buckwheat samples. Excellent resolution between (+)- and (−)-hyoscyamine was achieved, enabling reliable determination of the enantiomeric composition of naturally contaminated samples, together with low detection and quantification limits of 0.5 and 1 µg/kg, respectively (Table 3). A major contribution of this work was the demonstration that the extraction procedure did not induce enantiomerisation, thereby preserving the native stereochemical composition of the samples. Notably, only the naturally occurring (−)-hyoscyamine was detected in the raw seed materials, confirming the stereoselective biosynthesis of this alkaloid in plants and supporting EFSA considerations regarding the predominance of the natural enantiomer in food contamination events [16,28]. In addition, *D. stramonium* seeds contained substantially higher concentrations of (−)-hyoscyamine than *Brugmansia* seeds (1454 and 327 mg/kg, respectively). Even low levels of seed contamination resulted in measurable concentrations of the alkaloid in buckwheat samples, reaching 170 µg/kg and 100 µg/kg following contamination with *D. stramonium* and *Brugmansia* seeds, respectively [28]. These findings demonstrate the high transfer potential of naturally occurring (−)-hyoscyamine to food products and highlight the value of enantiomer-specific analysis for assessing contamination patterns and preserving information on the natural stereochemical composition of TAs in food matrices [16,28]. Furthermore, the authors evaluated the influence of pH and temperature on atropine racemisation, concluding that significant enantiomerisation occurred only under strongly alkaline conditions (pH 9) and elevated temperatures (80 °C). These findings provided valuable information for the development of analytical methodologies aimed at preserving the natural enantiomeric composition of TAs during sample preparation and analysis [28].

More recently, Vera-Baquero et al. [29] developed an HPLC-MS/MS method for the enantiomeric determination of (+)- and (−)-hyoscyamine using a polysaccharide-based CSP, namely Chiralpak^®^ AY-3 (Table 2). The final method provided Rs = 1.59 and method quantification limits of 14.8 and 15.3 µg/L in the extract for (+)- and (−)-hyoscyamine, respectively, corresponding to 0.089 and 0.092 µg/L in the original infusion after SPE preconcentration. The method showed matrix effects of 83% and 104% in representative infusion matrices and recoveries of approximately 88–93%, demonstrating suitability for trace analysis in real food samples [29].

Particular attention was devoted to evaluating the preservation of hyoscyamine enantiomeric integrity during sample preparation. Although the chromatographic conditions alone did not produce significant racemisation, measurable conversion of (−)-hyoscyamine into its (+)-enantiomer (below 20%) was observed during SPE, which was attributed to the alkaline conditions used in the ammonia-elution step. These findings are consistent with those reported by Marín-Sáez et al. [28]. To account for this sample-preparation effect, correction factors were applied to ensure reliable enantiomer-specific quantification. This result highlights the importance of assessing stereochemical changes throughout the entire analytical workflow, as adequate chromatographic resolution and MS sensitivity do not, in themselves, guarantee preservation of the original enantiomeric composition [29].

The applicability of the method was demonstrated through the chiral analysis of hyoscyamine enantiomers in commercial baby herbal infusions. The SPE preconcentration step proved essential for achieving the low quantification limits required for the determination of TAs at concentrations relevant to current food safety regulations in these samples. Hyoscyamine contamination was detected in several samples, with (−)-hyoscyamine being the predominant enantiomer in all positive samples. Among the instant infusions analysed, one sample contained (−)-hyoscyamine at 0.075 µg/L, whereas another contained both enantiomers, with concentrations of 0.21 and 0.09 µg/L for (−)- and (+)-hyoscyamine, respectively. This corresponded to an enantiomeric distribution of approximately 70% (−)-hyoscyamine and 30% (+)-hyoscyamine, suggesting a deviation from the natural enantiomeric composition expected in plant-derived materials. Importantly, the total hyoscyamine concentration in this sample (0.30 µg/L) exceeded the maximum level of 0.20 µg/L established for atropine in liquid herbal infusions. In contrast, for infusions prepared from dried herbal materials, hyoscyamine enantiomers were detected in only one sample, with concentrations of both enantiomers remaining below the method quantification limits [29].

These results highlight the value of enantiomer-specific determination in food safety studies. While conventional methods based on total atropine content provide information on overall contamination levels, chiral methodologies offer additional insight into the enantiomeric composition of samples, facilitating the detection of possible racemisation processes and deviations from the natural stereochemical profile expected for plant-derived contaminants. Furthermore, the combination of SPE preconcentration and sensitive HPLC-MS/MS detection enabled compliance with the stringent analytical requirements established by current European legislation for infant foods, while providing more comprehensive information for exposure assessment and regulatory monitoring of TA contamination [16,20,29].

## 6. Conclusions and Future Trends

The reviewed literature indicates that successful chiral HPLC analysis of TA depends less on identifying a universally superior CSP than on matching the selector, chromatographic mode, and mobile-phase composition to the target analyte and detection system. Nevertheless, several practical starting points emerge from the available evidence. For polysaccharide-based CSPs, NP conditions using alcohol modifiers, particularly EtOH, with low concentrations of DEA have proved useful for selected TAs, whereas RP methods combining MeOH with volatile aqueous buffers, such as ammonium bicarbonate, have enabled the separation of atropine and homatropine. For CD CSPs, MeOH–water mixtures containing TEAA provide another useful approach. These conditions are starting points for method development rather than universal optima; systematic screening remains necessary because changes in the analyte, selector, or mobile phase can substantially alter the separation.

A second key conclusion is that maximising enantioresolution in isolation is not sufficient for an effective analytical method. The best conditions must provide adequate separation while maintaining suitable retention, analysis time, and detector performance. This is particularly relevant to HPLC-MS/MS, where amine additives that improve chromatographic performance may also suppress electrospray ionisation. Mobile-phase optimisation should therefore consider chromatographic and detection requirements simultaneously, and final performance must be confirmed in the intended matrix rather than inferred from standard solutions alone.

The reliability of enantiomer-specific measurements also depends on preserving the original enantiomeric composition throughout extraction, clean-up, storage, and analysis. Because alkaline conditions, temperature, and exposure time can promote enantiomerisation in some circumstances, stereochemical stability should be assessed as part of method validation. Such assessment must distinguish genuine enantiomerisation from degradation reactions, including hydrolysis, that reduce the concentration of the parent alkaloid without necessarily generating the opposite enantiomer.

Future work should focus on validating these analytical strategies across a wider range of food matrices, improving the consistency with which chromatographic and validation parameters are reported, and establishing procedures to assess enantiomeric integrity throughout the analytical workflow. More interlaboratory studies and suitable enantiomerically characterised reference materials are also needed. Ultimately, reliable determination of individual TA enantiomers, combined with more extensive occurrence data in foods, will help clarify whether measured enantiomeric profiles reflect the original composition of contaminated products or changes introduced during processing and analysis, thereby strengthening exposure and risk assessments.

## Figures and Tables

**Table 1:** Main chiral tropane alkaloids and analytical relevance.

TA	Stereochemistry	Natural Occurrence	Pharmaceutical/Toxicological Relevance	Analytical Challenge
Hyoscyamine/atropine	Hyoscyamine contains one stereogenic centre in the tropic acid moiety. Natural biosynthesis predominantly yields (−)-hyoscyamine, whereas atropine corresponds to the racemic form, (±)-hyoscyamine.	Mainly found in *Solanaceae*, particularly in genera such as *Hyoscyamus*, *Atropa*, *Brugmansia*, and *Datura*.	Important antimuscarinic alkaloid used therapeutically; atropine is also a relevant contaminant in plant-derived foods and is associated with anticholinergic toxicity.	Enantiomeric determination is required to distinguish the naturally occurring (−)-enantiomer from the (+)-enantiomer and to assess possible changes in enantiomeric composition caused by racemisation during processing or analysis.
Scopolamine	Scopolamine is a chiral tropane alkaloid occurring predominantly as the naturally occurring (−)-enantiomer.		Used therapeutically for its anticholinergic properties and is also an important food contaminant because of accidental co-harvesting of TA-producing plants.	Enantioselective determination is relevant for characterising its natural stereochemical composition and for the analysis of contaminated food matrices.
Homatropine	Commercial homatropine is generally used as a racemic mixture and is structurally related to atropine and scopolamine.	Mainly encountered as a pharmaceutical compound rather than as a major naturally occurring TA.	Anticholinergic compound mainly used in ophthalmic preparations.	Separation of its enantiomers is required for stereochemical characterisation of the racemic pharmaceutical compound and illustrates the applicability of CSP-based methods to structurally related tropane derivatives.
Anisodamine	Contains two stereogenic centres. Natural anisodamine occurs predominantly as the (6S,2S) isomer, whereas synthetic anisodamine may contain four stereoisomers: (6S,2S)-, (6R,2R)-, (6S,2R)-, and (6R,2S)-anisodamine.	Originally isolated from *Anisodus tanguticus* (Maxim.).	Used therapeutically in the treatment of circulatory disorders, septic shock, and organophosphate poisoning. Different stereoisomers may exhibit distinct pharmacological activities.	The presence of multiple stereoisomers makes selective chromatographic separation and characterisation particularly challenging.
Littorine	Naturally occurring littorine is predominantly the (−)-enantiomer; its chirality originates from the stereogenic centre in the phenyllactate-derived side chain.	Found in *Anthocercis littorea* Labill. and involved in the biosynthetic pathway leading to hyoscyamine and scopolamine.	Primarily relevant as a biosynthetic intermediate in the formation of pharmacologically important TAs.	Its stereochemical characterisation is relevant for understanding TA biosynthesis and for the enantioselective analysis of structurally related tropane alkaloids.

**Table 3:** Summary of sample preparation procedures used for the chiral separation of tropane alkaloids (TAs) in pharmaceutical, biological, and food samples by liquid chromatographic techniques, based on articles published between 2010 and 2026, including limits of detection (LOD) and quantification (LOQ), recovery values, and the main application results.

Analytes	Sample	Sample Preparation	Recovery (%)	Results Achieved	[Ref.]
Pharmaceutical drugs					
Atropine and (1RS,2SR)-(±)-ephedrine	Atropine eye drop solution.	Eye drop solutions lyophilised were dissolving in MeOH followed by dilution in MeOH/H_2_O (60:40, *v*/*v*) to obtain concentrations of 1 mg/mL for analytical separation and 1–5 mg/mL for preparative separation.	94.3–98.9	The study successfully developed a method for enantiomerisation and semipreparative enantioseparation of atropine and (1RS,2SR)-(±)-ephedrine using a polyacrylamide CSP. The method proved effective for determining enantiomeric composition in pharmaceutical dosage forms, enhancing the efficacy of atropine as an antidote, and ensuring chiral integrity of labile molecules under ambient conditions without external stimuli.	[36]
Atropine and zopiclone	Isopto-atropine ophthalmic solution and Hypnor tablet.	Isopto-atropine ophthalmic solution was diluted with mobile phase, and Hypnor tablet was dissolved with ACN before analysis.	98.83–101.51	The study developed a method for direct enantioseparation of hyoscyamine sulfate enantiomers and zopiclone using a chiral AGP column. The method proved effective for determining enantiomeric impurities in pharmaceutical preparations, with thermodynamic studies showing enantioseparation driven by enthalpy and entropy effects.	[34]
Biological samples					
Atropine	Human plasma and rabbit serum.	Incubation of plasma with human serum (without atropinesterase) and rabbit serum (with atropinesterase), followed by protein precipitation with ACN and dilution of the supernatant before analysis.	93–101	The enantioselective assay is a reliable tool for quantifying hyoscyamine enantiomers in plasma, allowing the monitoring of enantioselective pharmacokinetics. The main drawback is potential matrix interferences inhibiting the atropinesterase-mediated hydrolysis of (-)-hyoscyamine.	[35]
Food samples					
Tropine, atropine, scopolamine, homatropine, anisodamine, and 2 glycoalkaloids	Animal feed and food (wheat, rye, maize, mixed grain flour, maize flour, biscuits, and crackers).	Grain samples were ground before dSPE with 0.5% FA in ACN/H_2_O and a mixture of MgSO_4_, NaCl and Na_3_C_6_H_5_O_7_. For oily matrix, MSPD C18 material was added to the extract as a clean-up step. Then they were shaken and centrifuged. Finally, an aliquot was evaporated to dryness under a stream of nitrogen. The residues were dissolved in a MeOH/ACN (50:50, *v*/*v*), sonicated in an ultrasonicator and filtered before injection.	61–111	The developed HPLC-MS/MS method employing a Vancomycin CSP allows for the simultaneous identification and quantification of tropane alkaloids and glycoalkaloids in various crops with high sensitivity, accuracy, and precision, making it suitable for routine analysis in food and feed safety assessments.	[32]
Atropine	*Datura stramonium*, *Brugmansia arborea* seeds and buckwheat samples (*Fagopyrum esculentum*).	Matrix extraction was based on a QuEChERS method, using water and ACN with AcOH. Then, Na_2_SO_4_ and NH_4_C_2_H_3_O_2_ were added to the mixture and vortex. The mixture was centrifuged and the supernatant was cleaned with PSA and GCB. Finally, the supernatant was filtered and diluted with EtOH (50/50, *v*/*v*) prior analysis.	N.S.	This study developed a simple method for the enantio-quantification of (±)-hyoscyamine in *Datura stramonium* and Brugmansia Arborea seeds and contaminated buckwheat with low detection and quantification limits employing an amylose-based CSP. The method was applied to evaluate the racemisation of (−)-hyoscyamine in (+)-hyoscyamine under different pH and temperature conditions, finding that racemisation occurs at high temperatures and basic pH. The method allows analysis without racemisation and can be applied to real samples.	[28]
Atropine	Baby herbal infusions: soluble plant extracts for instant infusions and dried plant mixtures in teabag format.	Instant infusions are reconstituted in H_2_O and tea bag infusions are prepared with boiling water. The pH is adjusted to 2.5, and the supernatant is filtered before the SPE performed with 50 mg of HMS-SO_3_^−^(co) material: conditioning with MeOH and 1% FA in H_2_O, extract loading, washing with MeOH/H_2_O/FA, 75/25/1 (*v*/*v*/*v*), and elution with 0.5% ammonia in MeOH. The eluate was evaporated to dryness and reconstituted with EtOH for analysis.	94–99	The developed method effectively resolves hyoscyamine enantiomers employing an amylose-based CSP and is suitable for monitoring these compounds in baby herbal infusions. The method is efficient, with high recoveries, and meets regulatory requirements for tropane alkaloid content in baby herbal infusions.	[29]

ACN: acetonitrile; AcOH: acetic acid; dSPE: dispersive solid phase extraction; EtOH: ethanol; FA: formic acid; GCB: graphitised carbon black; H_2_O: water; LOD: limit of detection; LOQ: limit of quantification; MeOH: methanol; MSPD: matrix solid-phase dispersion; PSA: primary secondary amine; QuEChERS: quick, easy, cheap, effective, rugged, and safe; SPE: solid-phase extraction.

## Data Availability

No new data were created or analysed in this study. The original contributions presented in this study are included in the article. Further inquiries can be directed to the corresponding authors.

## Abbreviations

The following abbreviations are used in this manuscript:

ACN	acetonitrile
AcOH	acetic acid
AGP	α1-acid glycoprotein
CD	cyclodextrins
CMPA	chiral mobile phase additive
CSP	chiral stationary phase
DAD	diode array detector
DEA	diethylamine
DEtOHA	diethanolamine
DNPCDSCP	native-perphenylcarbamoylated CD CSP
dSPE	dispersive solid phase extraction
EC	European Commission
EFSA	European Food Safety Authority
EtOH	ethanol
EU	European Union
FA	formic acid
FDA	Food and Drug Administration
GAC	Green Analytical Chemistry
GCB	graphitised carbon black
Hex	hexane
HPLC	high-performance liquid chromatography
ILs	ionic liquids
i-PrOH	isopropanol
LLE	liquid-liquid extraction
LOD	limit of detection
LOQ	limit of quantification
MeOH	methanol
MQL	method quantification limit
MS	mass spectrometry
MS/MS	tandem mass spectrometry
MSPD	matrix solid-phase dispersion
NP	normal phase
PO	polar organic phase
PrOH	propanol
PSA	primary secondary amine
QuEChERS	quick, easy, cheap, effective, rugged, and safe
RP	reversed phase
Rs	chiral resolution
Rt	retention time
S/N	signal-to-noise ratio
SCX	strong cation exchange
SLE	solid-liquid extraction
SPE	solid-phase extraction
SPP	surface porous particles
TAs	tropane alkaloids
TEA	triethylamine
TEAA	triethylammonium acetate buffer
TFA	trifluoroacetic acid
UV	ultraviolet
α	selectivity factor
